# Malaria epidemiology in the Ahafo area of Ghana

**DOI:** 10.1186/1475-2875-10-211

**Published:** 2011-07-29

**Authors:** Kwaku P Asante, Charles Zandoh, Dominic B Dery, Charles Brown, George Adjei, Yaw Antwi-Dadzie, Martin Adjuik, Kofi Tchum, David Dosoo, Seeba Amenga-Etego, Christine Mensah, Kwabena B Owusu-Sekyere, Chris Anderson, Gary Krieger, Seth Owusu-Agyei

**Affiliations:** 1 Kintampo Health Research Centre, Ghana Health Service, Ministry of Health, P. O. Box 200, Kintampo, Ghana; 2 College of Health Sciences, University of Ghana, Legon, Ghana; 3 Newmont Ghana Gold Limited C825/26 Lagos Avenue East, Legon, Accra, Ghana; 4 INDEPTH Network Secretariat, 11 Mensah Wood Street, East Legon P. O. Box KD 213, Kanda, Accra, Ghana; 5 HealthLink Consulting P.O. Box AN 6811, Accra-North Ghana; 6 Newfields 730 17th Street Suite 925 Denver, CO 80202, USA

## Abstract

**Background:**

*Plasmodium falciparum *malaria remains endemic in sub-Saharan Africa including Ghana. The epidemiology of malaria in special areas, such as mining areas needs to be monitored and controlled. Newmont Ghana Gold Limited is conducting mining activities in the Brong Ahafo Region of Ghana that may have an impact on the diseases such as malaria in the mining area.

**Methods:**

Prior to the start of mining activities, a cross-sectional survey was conducted in 2006/2007 to determine malaria epidemiology, including malaria parasitaemia and anaemia among children < 5 years and monthly malaria transmission in a mining area of Ghana.

**Results:**

A total of 1,671 households with a child less than five years were selected. About 50% of the household heads were males. The prevalence of any malaria parasitaemia was 22.8% (95% CI 20.8 - 24.9). *Plasmodium falciparum *represented 98.1% (95% CI 96.2 - 99.2) of parasitaemia. The geometric mean *P. falciparum *asexual parasite count was 1,602 (95% CI 1,140 - 2,252) and 1,195 (95% CI 985 - 1,449) among children < 24 months and ≥ 24 months respectively. Health insurance membership (OR 0.60, 95% CI 0.45 - 0.80, p = 0.001) and the least poor (OR 0.57, 95% CI 0.37 - 0.90, p = 0.001) were protected against malaria parasitaemia. The prevalence of anaemia was high among children < 24 months compared to children ≥ 24 months (44.1% (95% CI 40.0 - 48.3) and 23.8% (95% CI 21.2 - 26.5) respectively. About 69% (95% CI 66.3 - 70.9) of households own at least one ITN. The highest EIRs were record in May 2007 (669 *ib/p/m*) and June 2007 (826 *ib/p/m*). The EIR of *Anopheles gambiae *were generally higher than *Anopheles funestus*.

**Conclusion:**

The baseline malaria epidemiology suggests a high malaria transmission in the mining area prior to the start of mining activities. Efforts at controlling malaria in this mining area have been intensified but could be enhanced with increased resources and partnerships between the government and the private sector.

## Background

*Plasmodium falciparum *malaria remains endemic in sub-Saharan Africa [1]. In Ghana, the burden of malaria remains high with about 323 per 1,000 cases reported among children < 5 years in 2008; and there is limited evidence of a decrease in recent years (2002 - 2008) [2]. Malaria studies carried out in the middle belt of Ghana report of high transmission of approximately 269 infective bites per person per year and a parasite prevalence of not less than 50% at all times in the year among children < 5 years [3]. The burden of malaria in Ghana and other endemic countries will continue to result in anaemia, cerebral malaria, and severe malaria, if the use of currently available tools for malaria interventions such as rapid diagnosis and appropriate treatment, use of insecticide-treated nets (ITNs) and indoor residual spraying are not intensified as part of the strategies to control malaria. A complex matrix of factors could mitigate the successful use of the available tools; of special note are access to health care, household socioeconomic status, parental formal education, health education about malaria control and nutritional status of individuals. However, there have been reports of encouraging malaria control efforts in some countries in sub-Saharan Africa, including Zanzibar [4], Kenya [5,6] and Tanzania [7], which can be replicated in other African countries given the resources from public and private partnerships and political commitment available recently.

Newmont Ghana Gold Limited, a mining company in Ghana[8] recognizes that its mining activities may have an impact on malaria and other public health diseases in the mining area. For example, mining activities could alter the breeding potential of mosquitoes as well as increase migration into the mining area and this could precipitate poor environmental factors that may lead to diseases including malaria. Alternatively, environmental alterations as a result of urbanization and the mining activities may decrease mosquito breeding sites and, therefore, the burden of malaria.

With this realization, the Ghana Health Service in partnership with Newmont Ghana Gold Limited conducted a household and malaria epidemiology study as a baseline prior to the start of mining activities of Newmont Ghana Gold Limited in 2006/2007 to plan and evaluate malaria control programme within the area. This effort to control malaria in a mining area builds on a similar partnership in another mining area in Obuasi, Ghana that has led to a successful malaria control programme in the area [9]. This baseline malaria epidemiological data will be used for monitoring and evaluating malaria interventions specifically targeted at the mining area. The Ahafo baseline data are being reported in further attempts to contribute to the body of data required for monitoring global malaria burden.

## Methods

### Study area

The study was carried out in four districts in the Brong Ahafo Region of Ghana; the Asutifi District (between latitudes 6°40' and 7°15' North and Longitudes 2°15' and 2°45' West), the Tano North and South (Tano N/S) Districts (between latitudes 7°00'N and 7°25' N and between longitudes 1°45 W and 2°30 W), and the Techiman municipality (between latitudes 7°31'N and 8°0' N and between longitudes 1°60 W and 2°00 W). The study areas are in the wet semi-equatorial forest zone where the mean annual rainfall is about 1,200 mm per annum. Farming of cash crops, such as Cocoa, Oil Palm, Coffee are the major economic activity in the study communities. All health facilities in the study area implement both clinical and public health services under the supervision of the Brong Ahafo Regional Health Directorate. Malaria is the leading cause of morbidity and mortality in the region. About eighty percent of residents in the study area belong to the Akan ethnicity.

### Study design and methods

**Figure 1:**
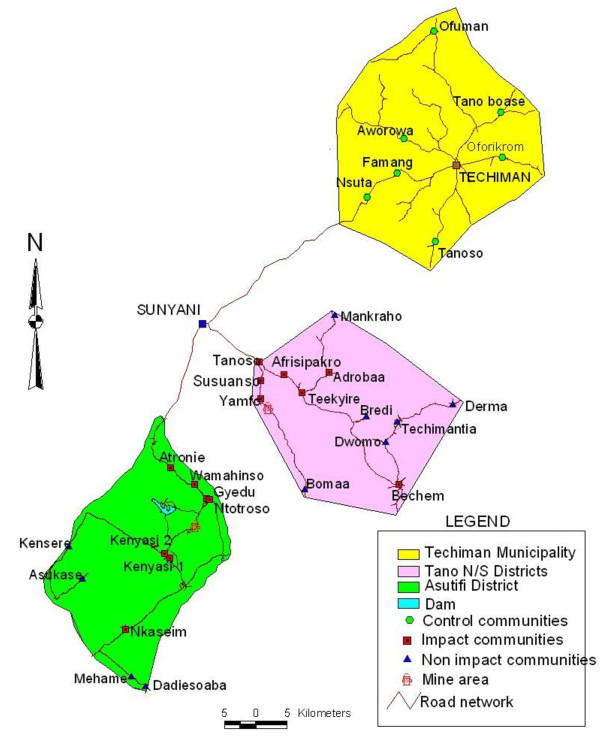
**Study area map**.

A household cross-sectional study was carried out between July 2006 and March 2007 to determine baseline household morbidity and malaria transmission prior to the start of mining activities in the area. The districts in the mining area were categorized into two; the Impact area involving communities within the Asutifi, and Tano N/S districts whose health and/or socio-economic indices are likely to be affected by mining activities being undertaken and located within approximately 25 Km radius from the mine sites. The Non-impact area representing communities within the Asutifi, Tano North or Tano South districts that are not likely to be affected by the mining activities and located more than 25 Km from the mines site (Figure 1). The Techiman municipality was purposively selected as a control area without mining activities but under the same local political and health administration as the other districts. The control area was selected for future comparisons after the beginning of mining activities. Within each area, communities were purposively selected based on geographical location to ensure representation of all locations within each area. Eleven (11) out of twenty (20); thirteen (13) out of nineteen (19) and eight (8) out of eighteen (18) communities were selected to represent the study areas in the Asutifi, Tano N/S and Techiman areas respectively.

### Household selection

All households in the selected communities were enumerated and encoded into a database. Each household member in the community including children less than five (5) years was given a unique identification number as part of the household morbidity survey. All households who were respondents for the household morbidity survey and with children less than five (5) years in the database formed the sampling frame. One child less than five (5) years was randomly selected per household using a computer software for anthropometric assessment, hematological and malaria parasitaemia.

Household ownership of ITNs and other household characteristics were assessed using a pre-tested structured questionnaire which were administered by trained fieldworkers. The quality of the ITNs was assessed by direct observation for holes.

Malaria parasitaemia was determined by collecting finger prick blood samples from participants to prepare thick and thin blood smears. The smears were stained with 10% Giemsa and air-dried. They were examined under the microscope using ×10 eyepieces and ×100 objective. Malaria parasites were counted against 200 white blood slides on the thick smear. A slide was declared negative only after 200 oil immersion fields of the thick smear had been examined. Parasites were expressed per microlitre, assuming a total white cell count of 8,000 per microlitre. Species identification was done using the thin smear. A sub-sample (10%) of the blood slides were randomly selected and sent to an independent expert microscopists for examination and the results compared with > 90% concordance.

Part of the blood from the finger prick was collected into Hemocue microcuvettes and haemoglobin levels were determined using a calibrated hemocue (HemoCue GmbH, Germany). All children found to be unwell, parasitaemic or anaemia during the survey were referred to a study clinician and treated.

Entomological surveys were carried out in the Asutifi and the Tano N/S districts from November 2006 to August 2007. The dry season period (Nov. 2006 - Feb. 2007) and wet season period (May 2007 - Aug. 2007) were purposefully planned to compare transmission with or without the influence of rainfall in a mining area. No entomology work was carried out in the Techiman district as similar studies were carried out in the Kintampo district, an adjacent district with similar rainfall pattern in 2004 [3]. Centre for Disease Control (CDC) light trap catches were performed concurrently on each night of mosquito collection in both impact and non-impact communities. Index persons were randomly selected from the Asutifi/Tano household enumeration database. A total of about 1,100 possible compounds were randomly selected from enumerated households in the Asutifi and Tano N/S districts to receive mosquito CDC light traps. The house of the "index" person was the house of choice to set traps. Traps were hung at the foot end adjacent to the "index" net of the "index" person. Untreated mosquito nets were provided to household members whose rooms were used on the night of trap setting to ensure they were protected while ensuring that mosquitos were not repelled. This method was approved by ethics committees. Four traps were set weekly and mosquitoes collected were chloroformed and morphologically identified using *Anopheline *morphological identification keys [10]. They were stored in 1.5 ml eppendorf tubes and transported on weekly basis to the Kintampo Health Research Centre laboratories for ELISA as per the procedures of Wirtz *et al *[11]. An average cut-off point of 0.2 nm absorbance wavelength was considered positive. Positive samples were re-tested and PCR analysis performed as described [11-13]. The entomologic inoculation rate (EIR) was calculated as the product of the proportion of *Anopheles *positive by ELISA also termed as sporozoite rate (SR) and the Human Biting Rate which is estimated as the geometric mean of *Anopheles *caught per CDC light traps set.

### Identification of *Anopheles gambiae *s.l

A total of 200 morphologically identified *Anopheles gambiae *s.l. specimens were further analysed by polymerase chain reaction (PCR) in order to establish members of the species complex and molecular forms composition [14].

### Data management and statistical analysis

All data collected in the field or the laboratory were logged for traceability, and then batched for double data entry and processing using Microsoft^® ^Visual FoxPro 6.0. All data management processes were done at the computer laboratory of the Kintampo Health Research Centre. Data analysis was done using StataCorp Stata 10, TX USA. Averages and proportions were used to provide descriptions of age- and parasite prevalence, density, infection rates and the proportions of parasite clones that persisted during the survey. Socio-economic status was calculated for each household using World Bank asset scores for Ghana, based on ownership of a television, radio, refrigerator, car, motorcycle, bicycle, farm, electricity in house, ceiling, floor cover, and number of individuals/sleeping room. The anthropometric indices height-for-age (HA), weight-for-age (WA), weight-for-height (WH), were expressed as Z-scores using the WHO Anthro for personal computers, Version 3.1, 2010: Software for assessing growth and development of the world's children. A child was identified as being malnourished if s/he scored < -2 in one of the HA, WA, or WH indices.

Malaria parasitaemia, anaemia (haemoglobin < 10 g/dL) and ITN usage were the main outcome variables. Odds ratios were calculated with their 95% confidence interval to assess the relationships between exposure and outcome variables using univariate and multivariate logistic regression models. The Wald's test and likelihood ratio test were used to estimate P values for exposure variables with two (2) levels and more than two (2) levels respectively in both the univariate and multivariate logistic regression models. P values less than 0.05 were considered statistically significant. Missing values were not included in the logistic regression models.

### Ethical approval

Ethical approval to conduct the study was granted by the Kintampo Health Research Centre Institutional Ethics Committee. Community entry involved explaining the study to key community opinion leaders followed by community durbars/meetings. At these meetings, the study's aims, objectives, risk and benefits were explained to all participants. A signed/thumb printed written consent was obtained from each respondent. All data collected are kept confidential.

## Results

### Household characteristics

A total of 1,671 households with a child less than five years were selected. Response rate was approximately 100%. About 50% of the household heads were males (Table 1).

**Table 1:** Household characteristics (N = 1671)

	n	%
**Gender of household head**		

Male	840	50.3

Female	818	49.0

Missing	13	0.8

**Age group of household head**		

15 - 29	182	10.9

30-39	454	27.2

40-49	445	26.6

50-59	229	13.7

60+	321	19.2

Missing	40	2.4

**Child age**		

< 24 months	609	36.4

≥ 24 months	1062	63.6

**Household head educational level**		

None	603	36.1

Primary	203	12.2

Middle/JHS	645	38.6

> middle/JHS	208	12.5

Missing	12	0.7

**Household socioeconomic status**		

Poorest	357	21.4

Second	333	19.9

Third	341	20.4

Fourth	333	19.9

Least poor	300	18.0

Missing	7	0.4

**Health Insurance membership**		

No	950	56.9

Yes	714	42.7

Missing	7	0.4

**Household size**		

≤ 6	712	42.6

> 6	952	57.0

Missing	7	0.4

**Area of residence**		

Asutifi impact	246	14.7

Asutifi non impact	150	9.0

Tano N/S impact	580	34.7

Tano N/S non impact	360	21.5

Techiman	328	19.6

Missing	7	0.4

JHS-Junior High School

Majority of household heads were between ages 30-39 years (27.8%) and 40-49 years (27.3). Majority of children (63.6%; 95% CI 61.2 - 65.9) in the households were ≥ 24 months. Health insurance coverage was 42.3%, (95% CI 40.5 - 45.3).

### Malaria parasitaemia

**Figure 2:**
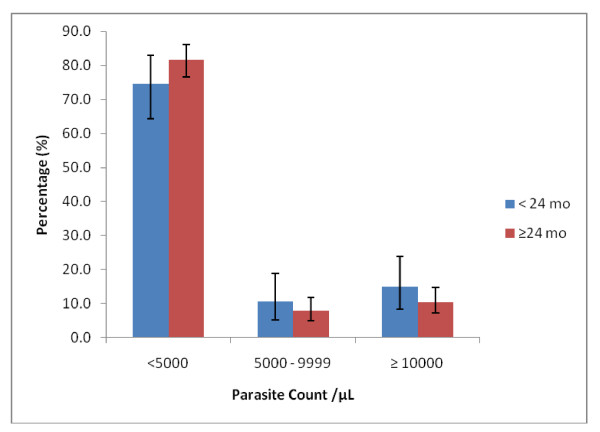
**Geometric Mean Parasite Density levels in the cohort ((< 24 mo, n = 94; > 24 mo, n = 279)**.

A total of 1,671 children less than five years were surveyed for malaria parasitaemia and anaemia. The prevalence of any malaria parasitaemia was 22.8% (95% CI 20.8 - 24.9). *Plasmodium falciparum *constituted 98.1% (95% CI 96.2 - 99.2) of parasitaemia. *Plasmodium malariae *was uncommon -1.9% (95% CI 0.81 - 3.8); no *Plasmodium ovale *or *Plasmodium vivax *was identified; there were no mixed infections. The geometric mean *P. falciparum *asexual parasite count was 1602 (95% CI 1,140 - 2,252) and 1,195 (95% CI 985 - 1,449) among children < 24 months and ≥ 24 months respectively. The proportion of children with parasite counts at various cut-off points was similar among the two age groups (Figure 2). Majority of the children (< 24 months: 74%, 95% CI 64.4 - 82.9; ≥ 24 months: 82.9%, 95% CI 76.7 - 86.1) had low parasite counts < 5,000/μL. A univariate logistic regression model analysis was fit prior to multivariate regression models. Malaria parasitaemia was significantly associated with the socioeconomic quintile (p < 0.001) with the strength of the association decreasing along the better socioeconomic quintiles. The least poor were significantly less likely to have malaria parasitaemia compared to the poor, OR 0.40 (95% CI 0.27 - 0.60) (Table 2). Health insurance membership protected against malaria parasitaemia (OR 0.51, 95% CI 0.40 - 0.66, p < 0.001). Household ITN ownership was weakly associated with malaria parasitaemia (OR 0.80, 95% CI 0.62 - 1.03, p = 0.08). Children ≥ 24 months were significantly more likely to have parasitaemia compared to children < 24 months (OR 1.95, 95% CI 1.50 - 2.53, p < 0.001) (Table 3). In the multivariate logistic regression model, there were strong evidence of associations between malaria parasitaemia and household membership with health insurance (p = 0.001); child age (p < 0.001) and anaemia (p < 0.001).

**Table 2:** Analysis of household attributes associated with malaria parasitaemia

Attribute	n/N (%)	OR^β^, (95% CI)	p	OR^γ^, (95% CI)	p
		**Unadjusted**		**Adjusted**	

**Gender of household head**					

Female	176/796 (22.1)	1	-	1	-

Male	188/827 (22.7)	1.04 (0.82 - 1.31)	0.764	0.99 (0.77 - 1.27)	0.954

**Age group of household head**					

15 - 29	39/180 (21.7)	1	-	1	-

30 - 39	74/447 (16.6)	0.71 (0.46 - 1.10)	0.003*	0.75 (0.47 - 1.20)	0.042*

40 - 49	107/428 (25.0)	1.21 (0.79 - 1.83)		1.26 (0.79 - 2.03)	

50 - 59	65/225 (28.9)	1.47 (0.93 - 2.32)		1.38 (0.82 - 2.34)	

60+	71/317 (22.4)	1.04 (0.67 - 1.62)		1.12 (0.66 - 1.91)	

**Household head educational level**					

None	164/586 (28.0)	1	-	1	-

Primary	42/200 (21.0)	0.68 (0.47 - 1.01)	< 0.001*	0.78 (0.51 - 1.18)	0.499*

Middle/JHS	125/631 (19.8)	0.64 (0.49 - 0.83)		0.80 (0.58 - 1.10)	

> middle/JHS	31/207 (15.0)	0.45 (0.30 - 0.69)		0.86 (0.52 - 1.41)	

**Household socioeconomic status**					

Poorest	103/348 (29.6)	1	-	1	-

Second	81/323 (25.1)	0.80 (0.57 - 1.12)	< 0.001*	0.88 (0.60 - 1.27)	0.047*

Third	83/334 (24.9)	0.79 (0.56 - 1.1)		0.91 (0.61 - 1.34)	

Fourth	52/327 (15.9)	0.45 (0.31 - 0.65)		0.59 (0.38 - 0.92)	

Least poor	43/296 (14.5)	0.40 (0.27 - 0.60)		0.56 (0.35 - 0.91)	

**Member of Health Insurance**					

No	250/927 (27.0)	1	-	1	-

Yes	112/701 (16.0)	0.51 (0.40 - 0.66)	< 0.001	0. 60 (0.45 - 0.80)	0.001

**Household ITN ownership**					

No	124/498 (24.9)	1	-	1	-

Yes	232/1107 (21.0)	0.80 (0.62 - 1.03)	0.079	0.81 (0.62 - 1.07)	0.147

**Household size**					

≤ 6	130/696 (18.7)	1	-	1	-

> 6	232/932 (24.9)	1.44 (1.13 - 1.84)	0.003	1.15 (0.85 - 1.54)	0.364

**Area of residence**					

Asutifi impact	50/236 (21.2)	1	-	1	-

Asutifi non impact	34/148 (23.0)	1.11 (0.68 - 1.82)	< 0.001*	0.85 (0.49 - 1.47)	0.065*

Tano N/S impact	94/575 (16.4)	0.73 (0.50 - 1.07)		0.92 (0.60 - 1.40)	

Tano N/S non impact	103/358 (28.8)	1.50 (1.02 - 2.21)		1.43 (0.93 - 2.20)	

Techiman	81/311 (26.1)	1.31 (0.88 - 2.0)		1.27 (0.79 - 2.02)	

β-Univariate logistic regression odds ratio (OR); γ-multivariate logistic regression odds ratio (OR); CI-confidence interval; * -Likelihood ratio p-values; JHS-Junior High School

**Table 3:** Analysis of child attributes associated with malaria parasitaemia

Attribute	n/N (%)	OR^β^, (95% CI)	p	OR^γ^, (95% CI)	p
		**Unadjusted**		**Adjusted**	

**Child age**					

< 24 months	93/600 (15.5)	1	-	1	-

≥ 24 months	273/1035 (26.4)	1.95 (1.50 - 2.53)	< 0.001	2.44 (1.79, 3.34)	< 0.001

**Height for age z-score**					

Normal	302/1403 (21.5)	1	-	1	-

Abnormal	59/209 (28.2)	1.43 (1.03 - 1.99)	0.031	1.17 (0.79 - 1.73)	0.442

**Weight for age z-score**					

Normal	333/1519 (21.9)	1	-	1	-

Abnormal	30/98 (30.6)	1.57 (1.01 - 2.46)	0.047	1.54 (0.84 - 2.85)	0.166

**Weight for height z-score**					

Normal	340/1500 (22.7)	1	-	1	-

Abnormal	18/104 (17.3)	0.71 (0.42 - 1.20)	0.206	0.56 (0.30 - 1.05)	0.071

**Anaemia**					

Non-anaemic	216/1130 (19.1)	1	-	1	-

Anaemic	150/505 (29.7)	1.79 (1.40 - 2.28)	< 0.001	1.70 (1.30 - 2.22)	< 0.001

β-Univariate logistic regression odds ratio (OR); γ-multivariate logistic regression odds ratio (OR); CI-confidence interval

#### Anaemia

**Figure 3:**
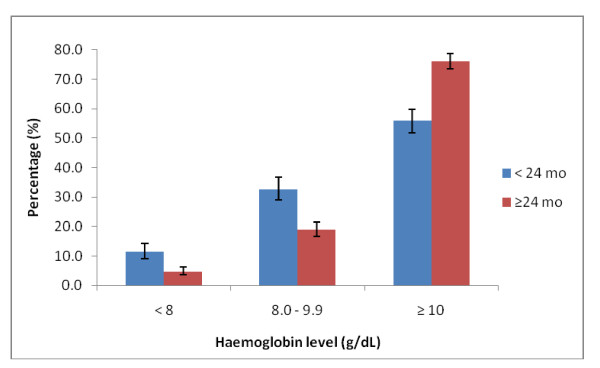
**Haemoglobin levels in the cohort of children (< 24 mo, n = 585; > 24 mo, n = 1035)**.

The prevalence of anaemia (haemoglobin < 10 g/dL) among all children < 5 years was 30.5% (95% CI 28.2 - 32.8). The prevalence of anaemia was high among children < 24 months compared to children ≥ 24 months 44.1% (95% CI 40.0 - 48.3) and 23.8% (95% CI 21.2 - 26.5) respectively. About 32.7% (95% CI 28.9 - 36.7) of the children < 24 months and 19.0% (95% CI 16.6 - 21.5) of children > = 24 months had haemoglobin levels between 8.0 - 9.9 g/dL (Figure 3).

In the univariate logistic regression model, children living in households with a male as a household head were significantly more likely to be anaemic compared to children living in households with females as household heads (OR 1.30; 95% CI 1.05 - 1.60, p = 0.016); childhood anaemia was less likely among household heads with characteristics, such as educational level > middle/Junior high school (OR 0.61; 95% CI 0.43 - 0.87, p = 0.029), the least poor socioeconomic status (OR 0.57 95% CI 0.40-0.80, p = 0.001) and household ITN ownership (OR 0.75 95% CI 0.61-0.93, p = 0.010). There was however no significant association between childhood anaemia and household health insurance membership or household size (Table 4). Children ≥ 24 months were significantly less likely to be anaemic compared to children < 24 months (OR 0.41 95% CI 0.33-0.51, p < 0.001) (Table 5). Attributes such as sex and age of household head, area of residence and child age remain significant in their relationship with anaemia in the multivariate logistic regression model.

**Table 4:** Analysis of household attributes associated with anaemia

Attribute	n/N (%)	OR^β^, (95% CI)	p	OR^γ^, (95% CI)	p
		**Unadjusted**		**Adjusted**	

**Gender of household head**					

Female	226/818 (27.6)	1	-	1	-

Male	278/840 (33.1)	1.30 (1.05 - 1.60)	0.016	1.28 (1.01 - 1. 61)	0.038

**Age group of household head**					

15 - 29	78/182 (42.9)	1	-	1	-

30 - 39	122/454 (26.9)	0.49 (0.34 - 0.70)	0.001*	0.55 (0.37 - 0.82)	0.024*

40 - 49	120/445 (27.0)	0.49 (0.34 - 0.71)		0.56 (0.37 - 0.86)	

50 - 59	78/229 (34.1)	0.69 (0.46 - 1.03)		0.64 (0.40 - 1.03)	

60+	97/321 (30.2)	0.58 (0.40 - 0.84)		0.49 (0.31 - 0.80)	

**Household head educational level**					

None	206/603 (34.2)	1	-	1	-

Primary	63/203 (31.0)	0.87 (0.62 - 1.22)	0.029*	0.72 (0.49 - 1.06)	0.080*

Middle/JHS	185/645 (28.7)	0.78 (0.61 - 0.98)		0.70 (0.52 - 0.95)	

> middle/JHS	50/208 (24.0)	0.61 (0.43 - 0.87)		0.84 (0.40 - 0.99)	

**Household socioeconomic status**					

Poorest	123/357 (34.5)	1	-	1	-

Second	120/333 (36.0)	1.07 (0.78 - 1.47)	0.001*	1.17 (0.82 - 1.68)	0.191*

Third	92/341 (27.0)	0.70 (0.51 - 0.97)		0.83 (0.57 - 1.22)	

Fourth	102/333 (30.6)	0.84 (0.61 - 1.16)		0.99 (0.67 - 1.46)	

Least poor	69/300 (23.0)	0.57 (0.40 - 0.80)		0.74 (0.48 - 1.14)	

**Member of Health Insurance**					

No	250/927 (27.0)	1	-	1	-

Yes	112/701 (16.0)	0.51 (0.40 - 0.66)	0.010	1.00 (0.77 - 1.30)	0.971

**Household ITN ownership**					

No	313/950 (33.0)	1	-	1	-

Yes	193/714 (27.0)	0.75 (0.61 - 0.93)	0.010	0.91 (0.69 - 1.20)	0.514

**Household size**					

≤ 6	201/712 (28.2)	1	-	1	-

> 6	305/952 (32.0)	1.20 (0.97 - 1.48)	0.095	1.28 (0.97 - 1.68)	0.080

**Area of residence**					

Asutifi impact	84/246 (34.2)	1	-	1	-

Asutifi non impact	86/150 (57.3)	2.59 (1.71 - 3.93)	< 0.001*	2.22 (1.38 - 3.55)	< 0.001*

Tano N/S impact	115/580 (19.8)	0.48 (0.34 - 0.67)		0.47 (0.32 - 0.68)	

Tano N/S non impact	139/360 (38.6)	1.21 (0.86 - 1.70)		0.97 (0.66 - 1.43)	

Techiman	82/328 (25.0)	0.64 (0.45 - 0.92)		0.49 (0.32 - 0.75)	

β-Univariate logistic regression odds ratio (OR); γ-multivariate logistic regression odds ratio (OR); CI-confidence interval; * -Likelihood ratio p-values; JHS-Junior High School

**Table 5:** Analysis of child attributes associated with anaemia

Attribute	n/N (%)	OR^β^, (95% CI)	p	OR^γ^, (95% CI)	p
		**Unadjusted**		**Adjusted**	

**Child age**					

< 24 months	259/609 (42.5)	1	-	1	-

≥ 24 months	247/1062 (23.3)	0.41 (0.33 - 0.51)	< 0.001	0.32 (0.25, 0.42)	< 0.001

**Height for age z-score**					

Normal	424/1429 (29.7)	1	-	1	-

Abnormal	76/211 (26.0)	1.33 (0.99 - 1.81)	0.062	1.62 (1.11 - 2.37)	0.012

**Weight for age z-score**					

Normal	457/1550 (29.5)	1	-	1	-

Abnormal	43/99 (43.4)	1.84 (1.22 - 2.77)	0.004	1.52 (0.86 - 2.68)	0.147

**Weight for height z-score**					

Normal	456/1523 (29.9)	1	-	1	-

Abnormal	41/105 (39.1)	1.50 (0.99 - 2.25)	0.051	1.17 (0.70 - 1.98)	0.546

β-Univariate logistic regression odds ratio (OR); γ-multivariate logistic regression odds ratio (OR); CI-confidence interval

### ITN ownership

About 68.7% (95% CI 66.3 - 70.9) of households own at least one ITN. The median ITN per household was 2 (range 1 - 7). About 18.1% (95% CI 15.9 - 20.5) of ITNs observed for quality had holes in them. About 86.4% (95% CI 84.3 - 88.4) of household that owned ITNs had at least one person sleeping under an ITN the night before the interview. Majority of ITNs (64.9%, 95% CI 62.1 - 67.7) were obtained free of charge as part of a national campaign while 26.4% (95% CI 23.8 - 29.0) bought their ITNs on the open market at commercial prices. There was no significant association between household ITN ownership and household head characteristics such as sex and educational level in the univariate logistic regression model (Table 6). There was however a significant association between ITN ownership and attributes such as health insurance membership and area of residence that remained significant in the multivariate logistic regression models. Households with children ≥ 24 months were significantly less likely to own an ITN (Table 7)

**Table 6:** Analysis of household attributes associated with household ITN ownership

Attribute	n/N (%)	OR^β^, (95% CI)	p	OR^γ^, (95% CI)	p
		**Unadjusted**		**Adjusted**	

**Gender of household head**					

Female	561/804 (69.8)	1	-	1	-

Male	555/823 (67.4)	0.90 (0.73 - 1.11)	0.309	0.88 (0.70 - 1.10)	0.271

**Age group of household head**					

15 - 29	127/180 (70. 6)	1	-	1	-

30 - 39	327/446 (73.3)	1.15 (0.78 - 1.68)	0.018*	1.06 (0.70 - 1.60)	< 0.001*

40 - 49	303/439 (69.0)	0.93 (0.64 - 1.36)		0. 69 (0.45 - 1.06)	

50 - 59	140/227 (61.7)	0.67 (0.44 - 1.02)		0.43 (0.27 - 0.70)	

60+	205/316 (64.9)	0.77 (0.52 - 1.14)		0.47 (0.29 - 0.77)	

**Household head educational level**					

None	406/593 (68.5)	1	-	1	-

Primary	132/203 (65.0)	0.86 (0.61 - 1.20)	0.131*	0.91 (0.62 - 1.32)	0.402*

Middle/JHS	433/637 (68.0)	0.98 (0.77 - 1.24)		0.98 (0.73 - 1.32)	

> middle/JHS	152/202 (75.3)	1.40 (0.97 - 2.01)		1.35 (0.86 - 2.10)	

**Household socioeconomic status**					

Poorest	237/348 (68.1)	1	-	1	-

Second	214/330 (64.9)	0.86 (0.63 - 1.19)	0.013*	1.0 (0.70 - 1.43)	0.148*

Third	226/338 (66.9)	0.95 (0.69 - 1.30)		0.97 (0.67 - 1.41)	

Fourth	223/330 (67.6)	0.98 (0.71 - 1.35)		1.05 (0.72 - 1.55)	

Least poor	226/294 (76.9)	1.56 (1.09 - 2.21)		1.55 (1.01 - 2.38)	

**Member of Health Insurance**					

No	608/937 (64.9)	1	-	1	-

Yes	518/703 (73.7)	1.51 (1.22 - 1.88)	< 0.001	1.35 (1.04 - 1.74)	0.022

**Household size**					

≤ 6	462/697 (66.3)	1	-	1	-

> 6	664/943 (70.4)	1.21 (0.98 - 1.49)	0.075	1.37 (1.05 - 1.79)	0.020

**Area of residence**					

Asutifi impact	107/246 (43.5)	1	-	1	-

Asutifi non impact	80/150 (53.3)	1.48 (0.99 - 2.23)	< 0.001*	1.44 (0.91 - 2.27)	< 0.001*

Tano N/S impact	423/562 (75.3)	3.95 (2.88 - 5.43)		3.55 (2.53 - 4.99)	

Tano N/S non impact	268/359 (74.7)	3.83 (2.27 - 5.41)		3.90 (2.68 - 5.67)	

Techiman	248/323 (76.8)	4.30 (2.99 - 6.16)		4.84 (3.22 - 7.26)	

β-Univariate logistic regression odds ratio (OR); γ-multivariate logistic regression odds ratio (OR); CI-confidence interval; * -Likelihood ratio p-values; JHS-Junior High School

**Table 7:** Analysis of child attributes associated with household ITN ownership

Attribute	n/N (%)	OR^β^, (95% CI)	p	OR^γ^, (95% CI)	p
		**Unadjusted**		**Adjusted**	

**Child age**					

< 24 months	491/596 (82.3)	1	-	1	-

≥ 24 months	635/1044 (60.8)	0.33 (0.26 - 0.42)	< 0.001	0.26 (0.20 - 0.35)	< 0.001

**Height for age z-score**					

Normal	960/1399 (68. 6)	1	-	1	-

Abnormal	147/210 (70.0)	1.07 (0.78 - 1.46)	0.687	0.98 (0.68 - 1.42)	0.935

**Weight for age z-score**					

Normal	1042/1519 (68. 6)	1	-	1	-

Abnormal	71/99 (71.7)	1.16 (0.74 - 1.82)	0.517	0.95 (0.53 - 1.72)	0.877

**Weight for height z-score**					

Normal	1028/1494 (68.8)	1	-	1	-

Abnormal	73/103 (70.9)	1.10 (0.71 - 1.71)	0.661	0.95 (0.54 - 1.57)	0.767

**Anaemia**					

Non-anaemic	782/1138 (68.7)	1	-	1	-

Anaemic	344/502 (68.5)	0.99 (0.79 - 1.24)	0.939	1.24 (0.96 - 1.60)	0.104

β-Univariate logistic regression odds ratio (OR); γ-multivariate logistic regression odds ratio (OR); CI-confidence interval

### Mosquito abundance and entomological inoculation rates (EIRs)

A total of 5,257 mosquitoes were caught from 818 light traps set; *Anopheles gambiae *comprised of 64.1% (n = 3,371) of the mosquitoes caught, *Anopheles funestus *4.3% (n = 227) and non-*Anopheles *species 31.6% (n = 1,659) which were predominantly *Culex *and *Aedes *species of mosquitoes. A few *Mansonia *species were recorded in Asutifi (4) and Tano N/S districts (2).

**Figure 4:**
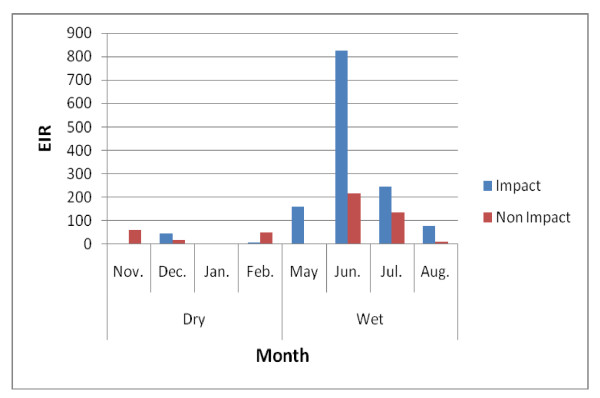
**Monthly EIRs in Asutifi district (November 2006-August 2007)**.

**Figure 5:**
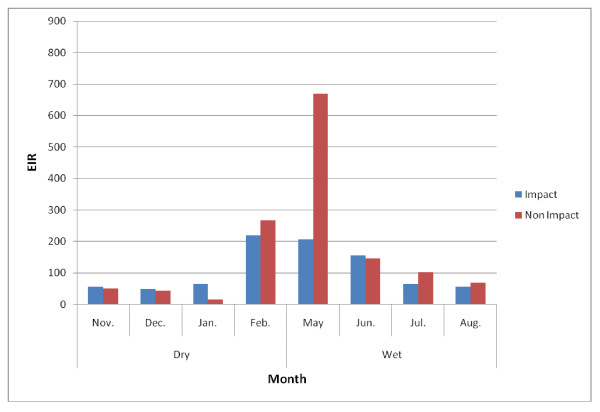
**Monthly EIRs in Tano N/S district (November 2006-August 2007)**.

EIRs for both *Anopheles gambiae *and *Anopheles funestus *were generally high. In the impact and non-impact areas of the Asutifi district, the median EIR was 46 *ib/p/m *(range 0-826 *ib/p/m*) and 51 *ib/p/m *(0 - 216) in the impact and non impact areas respectively. The highest was recorded in June 2007 at both impact and non impact areas respectively (Figure 4). EIRs in Tano N/S districts were also high, median 65 *ib/p/m *(range 48 - 219 *ib/p/m*) and 102 (range 14 - 669 *ib/p/m*) in the impact and non-impact area respectively with the highest in May 2007 (Figure 5).

**Figure 6:**
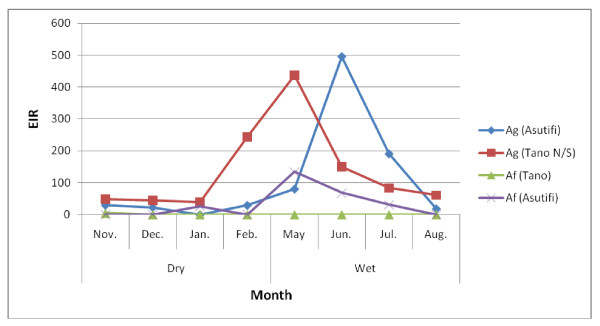
**Monthly EIRs by *Anopheles gambiae *and *Anopheles funestus *in Asutifi and Tano N/S districts (November 2006-August 2007)**. Ag = *Anopheles gambiae. Af = Anopheles funestus *(November 2006-August 2007)

The EIR of *Anopheles gambiae *were generally higher than *Anopheles funestus*. In Tano N/S alone, the median EIR for *Anopheles gambiae *alone was 73 *ib/p/m *(range: 39 - 438 *ib/p/m*) compared to *Anopheles funestus *median 0 *ib/p/m *(range 0 - 5 *ib/p/m*) alone (Figure 6).

### Polymerase chain reaction analysis

Mosquitoes randomly selected and amplified by PCR were all *An. gambiae sensu-stricto *as confirmed by the size of the amplified DNA fragment of 390 base pairs. One hundred out of the 200 mosquitoes identified as *An. gambiae s.s. *were randomly selected for the identification to the molecular form by enzyme digestion with *Hha*1. All 100 mosquitoes were S-forms shown by restriction analysis.

Two hundred mosquitoes were randomly selected to determine knockdown resistance (*kdr*) mutations. One hundred and ninety three out of the 200 selected mosquitoes (97%) possessed the *kdr *mutation (*kdr+*), whilst seven (3%) did not (*kdr-*).

## Discussion

The study assessed the epidemiology of malaria in a rural forest area of the Brong Ahafo region in Ghana. The prevalence of malaria parasitaemia and anaemia among children less than five years and malaria entomology were determined. Though renewed efforts in controlling malaria in sub-Saharan Africa has led to reports of decline in the burden of malaria in some parts of Africa [4-7,9], there is little evidence in Ghana to suggest a decline in out-patient attendance due to malaria and malaria mortality amidst the intensified delivery of malaria control interventions [2]. In this survey, a malaria epidemiology survey in a forest area of Ghana including a mining area was conducted to plan malaria control programmes in these areas.

The prevalence of *P. falciparum *among children gives an estimation of the burden of malaria in a particular area. In the study area, the prevalence of malaria parasitaemia was relatively low 22.8% (95% CI 20.8 - 24.9) compared to reports from other rural parts of Ghana; 58% in a forest savanna transitional area of Kintampo in 2004 [3]; between 55.5 - 69.3% in savanna area in northern Ghana between 2000 and 2002 [15,16]. The prevalence of malaria parasitaemia in the area was however comparable to reports in urban semi-humid areas - 12.8% - 37.8% in 2005 [17].

The relationship between malnutrition and malaria are inconsistent in the literature. Studies conducted in Ethiopia [18] and southern Ghana[19] showed no evidence of relation between malnutrition and anaemia contrary to studies conducted in urban Equatorial Guinea [20], in western Kenya [21] and reviews by Caulfield *et al *[22] that suggest a strong relationship between malnutrition and malaria. In this study, there was no association of malaria parasitaemia with malnutrition.

In 2003, the government of Ghana introduced the National Health Insurance Scheme with the aim of reducing financial constraints as a gap in accessing health. Household membership of the insurance scheme provides children to seek health in health facilities closest to them. Common illnesses, such as malaria, are treated without direct cash payment at the point of service. In this study, health insurance membership was protective against malaria parasitaemia but had no significant relationship with anaemia. Participants who subscribe to the health insurance may regularly seek health care and may have been treated for malaria thus, the observed protection of malaria parasitaemia by health insurance membership.

ITNs are known to prevent malaria infection by reducing human contact with the malaria vector and thus able to reduce all-cause mortality in children less than five years old by 17% in northern Ghana [23] and about 44% in Kenya [24]. ITN ownership has increased in recent times due to the support of local government and international donors such as USAID and their private partners [25]. In 2003, ITN ownership in Ghana was less than 10% but had increased to about 33% in 2008 [26]. In this study ITN ownership (at least one) was lowest in the Asutifi area (range 43% - 53.3%) compared to 76.8% in the Techiman area. This difference in household ITN ownership was due to mass distribution of ITNs to all households with children less than two years as part of Child Survival strategies throughout Ghana. The relatively low coverage in Asutifi is because this survey was carried out prior to the free distribution of treated bed-nets in the Child Survival Campaign. The coverage determined in the study area are similar to the coverage of 45.6% assessed in 2008 for the same area during the Ghana Health and Demographic Survey [26]. The coverage of ITNs could further be increased and maintained at the World Health Organization target of ≥80% if private companies such as Newmont Ghana Gold Limited and other partners could support the Ghana Health Service and the communities with additional ITNs.

Despite the benefits of ITNs in controlling malaria, its patronage has not been quite encouraging and its usage is for some reasons other than for malaria control. For instance, in Savalou, Benin mosquito nets were often seen as a means of protection against mosquitoes and other biting insects in order to sleep better rather than as a means of preventing malaria [27]. Several reasons including: lack of money or expensive ITN, unavailability of ITN, no provision for nets to fit sleeping space account for the low mosquito net usage. In this study, it was encouraging to note that ITN use was high; about 86.4% (95% CI 84.3 - 88.4) of household that owned ITNs had at least one person sleeping under an ITN the night before the interview. This is consistent with the high coverage of ITN use among children in the same area determined in 2008 [26]. Though there was a high ITN ownership and use, there was no evidence of protection against malaria parasitaemia after adjusting for age, anaemia, malnutrition and other household. This may be due to poor quality of ITNs (about 18% of household nets had holes) or improper use of ITNs.

One of the commonest causes of anaemia in sub-Saharan Africa is malaria. In this study, the prevalence of anaemia among all children < 5 years was moderately high 30.5% (95% CI 28.2 - 32.8) with children less than 24 months having a higher risk of anaemia. Although high, it is still lower than reported in other parts of Ghana and follows the same trend as malaria parasitaemia [3,15,16,28]. There are other causes of anaemia in children such as malnutrition, hookworm infection, and sickle cell disease but their contribution to anaemia in malaria endemic regions has been found to be minimal compared malaria [29,30]. In this study, there was a weak-association between abnormal height for age scores and anaemia; and a stronger association between malaria parasitaemia and anaemia after adjusting for age, ITN use and other household characteristics. Other common causes of anaemia such as hookworm infestation and sickle cell disease were not determined in this study.

There are few longitudinal malaria entomology studies carried out in mining communities in Ghana and other parts of the world. The abundance of mosquitoes in the Asutifi and Tano N/S districts differed slightly due to several contributing factors. Unlike Asutifi where the mosquito abundance was highest in the impact area, Tano experienced the reverse within the six month surveyed period. Though reasons for these differences in vector densities were not investigated, they may be attributed to environmental factors, such as changes in climate or vegetation, rainfall pattern, temperature; human factors such as human created breeding sites from urbanization; and intrinsic vector characteristics as previously reported [31,32].

The EIRs calculated in this survey were as high as the EIRs found in the Kintampo area (269 *ib/p/y*) two years prior to this survey and used as an estimate for the control. The high EIRs recorded occurred in months that experienced moderate rainfall; June-July in Asutifi and February-May in Tano N/S, which indicates the impact of rainfall in vector populations and their breeding potential. Moderate rainfall creates pockets of water which favours breeding of larvae unlike torrential rains which washes away larval breeding sites of the major malaria vectors in Africa [32]. As such, changes in vegetation or environment that promotes moderate rainfall could possibly begin to experience high malaria transmission if appropriate vector control activities are not planned. EIRs varied significantly between areas or communities and, therefore, control strategies need to be planned in concert with detail area vector indices such as abundance, speciation and EIRs. The low infectivity rate of *An. funestus *in this survey may probably be because a large proportion of young vectors with a low potential to transmit malaria may have been caught in the light traps; however this could not be confirmed since samples were not subjected to physiological age grading technique (parity detection) in this study. All *An. gambiae s. l*. analysed by PCR were of the S-molecular forms which is widespread in West Africa and other parts of Africa including the Kintampo area estimated for the control area [31-35].

The high percentage of *kdr*+ mutation reported in this study should prompt the malaria control programme to mount a surveillance on insecticide resistance existing especially since there could be an exacerbation due to the potential of cross-resistance between the insecticides used for farming and ITNs in this predominantly farming area.

At the time of the survey in 2006, the study area in the Asutifi and Tano N/S districts were being prepared for mining activities by Newmont Ghana Gold Limited. Part of the mining preparatory activities may include clearing away some forest vegetation that could potentially lead to clean water pools and subsequently change the breeding characteristics for mosquitoes that could transmit malaria and other vector borne diseases such as Yellow fever and Dengue if left unmanaged. This phenomenon of mosquito scourge associated with industrial activities was reported many years ago in the Panama Canal [36] and recently in the copper mines of DRC and Zambia [37,38]. In both cases, the industrial activity was significantly affected until an effective malaria control programme including environmental vector control was implemented. In the Zambian case, copper production increased significantly with declining burden of malaria, thus paying off the investment made in the malaria control. Additionally, a mining area has the potential of attracting migrant workers which increases the population with poor sanitation if uncontrolled.

This malaria survey was conducted as part of preparatory activities to control malaria and other potential vector borne diseases in the area. It demonstrates the mining company's commitment in controlling malaria in their area of operations while protecting its workforce to minimize cost due to healthcare and maximize productivity. Newmont Ghana Gold Limited and the Ghana Health Service have intensified malaria control activities in the area since the completion of the survey. These activities include in-door residual spraying, larviciding, free ITN distribution to employees (three ITNs per employee) and community members (two ITNs per household); refurbishment of a community health centre and training of health staff for accurate malaria diagnosis [39]. The impact of the interventions in the community is yet to be evaluated; however, a marked decrease (8% in 2006 to 1.8% in 2009) in malaria incidence among the mining work force has been noted [40]. Newmont Ghana Gold Limited's support to control malaria and other public health diseases, such as tuberculosis and HIV/AIDS, has been recognized by the Global Business Coalition on HIV/AIDS, Tuberculosis and Malaria in 2010 [40]. Similar private cooperate involvement in malaria control has successfully been supported by companies, such as Anglogold Ashanti in Obuasi area of Ghana [9], Konkola Copper Mine in Zambia [41] and Exxon Mobil in Angola, Cameroon, Chad, Equatorial Guinea and Nigeria [42].

## Conclusion

The malaria epidemiology suggests a high malaria transmission in the mining area prior to the start of mining activities. Efforts at controlling malaria in this mining area have been intensified but could be enhanced with increased resources and partnerships between government and the private sector. Periodic evaluations will be key to ascertain the impact of the malaria control activities currently ongoing in the mining area.

## Competing interests

The authors declare that they have no competing interests.

## Authors' contributions

KPA led in the proposal writing with contribution from CM, CZ, GK, KT, DD and SOA. KPA, CZ, DD, and KT coordinated the data collection during the study with support from YAD and CA on behalf of Newmont Ghana Gold Limited. Laboratory analysis was performed by DBD, CB, KT and DD. Data management and analysis was done by KPA, GD, MA, SAE and SOA. KPA wrote the manuscript with contribution from all authors. All authors read the final version of the manuscript.
